# Biomonitoring of bisphenol A concentrations in maternal and umbilical cord blood in regard to birth outcomes and adipokine expression: a birth cohort study in Taiwan

**DOI:** 10.1186/1476-069X-10-94

**Published:** 2011-11-03

**Authors:** Wei-Chun Chou, Jyh-Larng Chen, Chung-Fen Lin, Yi-Chun Chen, Feng-Cheng Shih, Chun-Yu Chuang

**Affiliations:** 1 Department of Biomedical Engineering and Environmental Sciences, National Tsing Hua University, Hsinchu, Taiwan; 2 Department of Environmental Engineering and Health, College of Health Science, Yuanpei University, Hsinchu, Taiwan; 3 Department of Health Care Administration, Chang Jung Christian University, Tainan, Taiwan

## Abstract

**Background:**

Bisphenol A (BPA) is a sealant and flux of plastic materials and has been determined to be an endocrine-disrupting chemical. Prenatal exposure to BPA can lead to substantial adverse effects on fetal growth and development. This study was conducted to assess BPA concentration in pregnant women and umbilical cord blood, and to investigate whether maternal BPA exposure affected fetal outcomes including lower birth weight (LBW), smaller size for gestational age (SGA), and high leptin (HLP) and low adiponectin (LAD) secretion.

**Methods:**

We measured the BPA levels of maternal blood (n = 97) and umbilical cord blood (n = 97) with a high-performance liquid chromatography/UV detector. The protein secretion of leptin and adiponectin were separately determined using enzyme-linked immunosorbent assay. A logistic regression was performed to estimate the effects of maternal exposure to BPA on LBW, SGA, and adverse action of adipokines in newborns.

**Results:**

The geometric means of BPA concentration in maternal blood and fetal cord blood were 2.5 ng/ml and 0.5 ng/ml, respectively. Elevated risks of LBW (OR 2.42, 95% confidence interval (CI) 1.72-3.36), SGA (OR 2.01, 95% CI 1.39-3.01), and adverse action of leptin (OR 1.67, 95% CI 1.12-2.25) and adiponectin (OR 1.25, 95% CI 1.52-3.97) were observed in male neonates in the highest quartile of maternal BPA exposure.

**Conclusions:**

Elevated prenatal BPA exposure increased the risk of LBW, SGA, and adverse actions of adipokines in neonates, especially in male infants. These results provide further evidence that maternal exposure is correlated with adverse birth outcomes.

## Background

Bisphenol A (2, 2-bis (4-hydroxyphenol) propane; BPA), a chemical compound found in plastic products, is being used increasingly in industrial manufacturing materials. Numerous reports state that BPA production was 2, 214, 000 metric tons worldwide per year in 2003 [1], and 3, 200, 000 tons in 2005 [2]. Because BPA is used to manufacture polycarbonate plastic, epoxy resins and certain dental sealants [3], humans are frequently exposed to BPA released from plastics and food cans in daily life [4]. Therefore, through these daily exposures BPA potentially affects human health.

Previous studies through analyses of BPA in the serum of pregnant women and in cord blood collected at birth have indicated that BPA accumulates early in fetuses [5,6]. Schonfelder et al. [7] stated that BPA levels vary: from 0.3 to 18.9 ng/ml (median 3.1 ng/ml) in maternal plasma, from 0.2 to 9.2 ng/ml (median 2.3 ng/ml) in fetal plasma, and from 1.0 to 104.9 ng/g (median 12.7 ng/ml) in the placenta. BPA can readily cross the placenta [8], and some *in vivo *experiments have demonstrated that BPA can cause adverse birth outcomes in offspring. Pregnant rats were orally administered BPA at a dose of 10 mg/kg/day resulting in a decreased number of neonates and decreased survival rate [9]. Female genital tracts developed abnormally when the dams were exposed to BPA through gavage [10] or osmotic pumps [11]. Pregnant rats exposed to a low dose of BPA (1 mg/L) in drinking water showed increased adipogenesis in females at weaning [12].

*In utero *or neonatal exposure to BPA can alter offspring phenotype by stably altering the epigenome, an effect that can be counteracted by maternal dietary supplements [13]. Male offspring were orally exposed at lower dosages of BPA (0.05 mg/kg/day), which led to a significant decrease in weight gain; however, offspring exposed to BPA treatment at a higher dosage (50 mg/kg/day) showed higher body weights than the controls [14].

Mechanistically, environmental BPA is a well-known endocrine-disrupting chemical that binds to estrogen receptors (ER) α and ERβ and results in competition with estrogen [15], while disrupting the folding, assembly, and shedding of many cellular proteins by targeting protein disulfide isomerase [16]. BPA can accumulate in adipose tissue. Fernandez et al. [17] detected an average 3.2 ng BPA/g fat and 8.2 ng chlorinated BPA/g fat in female adipose tissue. Olea et al. [18] found substantially higher levels in children. Moreover, BPA present in adipose tissue may alter the release of adiponectin and leptin, which could influence insulin resistance and increase susceptibility to obesity-associated diseases [19]. Additionally, BPA triggers adipocyte differentiation [20]. The treatment of BPA in mouse 3T3-L1 embryonic fibroblasts stimulates triglycerol accumulation in adipocytes and hepatocytes, and promotes preadipocytes to differentiate into mature adipocytes. The 3T3-L1 cells treated with BPA increase the levels of lipoprotein lipase and adipocyte-specific fatty acid binding protein (aP2) mRNAs. Therefore, BPA might mimic estrogen properties and alter adipokine release, thereby affecting fetal growth. Thus, this study examined the BPA distribution in maternal and umbilical cord blood, and its profile of adipokine protein. We also investigated the effects of prenatal BPA exposure on fetal birth outcomes including fetal development and hormone regulation.

## Methods

### Study subjects

One hundred and fifty-seven healthy pregnant women were recruited for this study between January 2006 and August 2007 at an obstetrics and gynecology clinic in Hsinchu County, Taiwan. All pregnant women consented to participate in this study, and the bio-sampling process was approved by the institutional review boards of National Tsing Hua University. One hundred and thirty-four subjects (response rate 85.3%) completed a self-reported questionnaire including physical characteristics (age, height, weight, occupation, cigarette smoking, betel quid chewing, and alcohol consumption), dietary habit, and disease history. Of the 97 mother-newborn pairs enrolled in the study (72.4%). 37 dropped out before delivery.

### Sample preparation

Maternal blood corresponding to umbilical cord blood samples were respectively collected in glass heparin tubes at full-term delivery. Plastics were excluded throughout the entire analytic procedure to avoid BPA contamination. Whole blood was centrifuged at 12, 000 rpm for 10 min to separate the plasma and corpuscles, and stored at -80°C until analysis. To the plasma fraction (500 μl) was added 100 μl of 0.01 M ammonium acetate buffer (pH 4.5; Riedel-de Haen, Seelze, Germany) and 4 ml mixture of n-hexane (HPLC grade; Echo Chemical, Miaoli, Taiwan) and diethyl ether (70:30 v/v, anhydrous; J.T. Baker, Phillipsburg, NJ). The samples were mixed for 5 seconds, vortexed for 10 minutes, immobilized for one minute, and then 8.71 μl of 9.187 M perchloric acid (purity 60-62%; Sigma-Aldrich, St. Louis, MO) was added. After centrifugation at 3, 000 rpm for 5 minutes, the organic layer was evaporated to dryness, and reconstituted with 100 μl of mobile phase (methanol:water 80:20 v/v) for BPA determination by a reverse-phase high performance liquid chromatography (HPLC).

### Chromatographic system and conditions

The BPA concentrations in plasma were determined using HPLC chromatography (D-7000) connected to a UV detector (L-7400) consisting of an autosampler (L-7200), a pump (L-2130) and a degasys (DG-2410) (Hitachi High Technologies America, Pleasanton, CA). HPLC conditions were as follows: 20 μl injection volume, Inertsil OctaDecylSilane (ODS)-3V column (5 μm, 250 mm × 4.6 mm; GL Sciences, Torrance, CA) fitted with a Metaguard Polaris C18 cartridge (5 μm, 4.6 mm; Varian, Lake Forest, CA) at room temperature, and mobile phase methanol:water (80:20 v/v) with a flow rate of 0.7 ml/min for 20-minute run time. The eluted peak of BPA (bis-(4-hydroxyphenyl)-propane, purity > 99%; Sigma-Aldrich, St. Louis, MO) was detected at 226 nm, and chromatographic data were analyzed by D-7000 multi-system software version 4.1. Both the initial standard stock solution, as well as the serial dilutions from the stock solution in methanol for the HPLC study, were 0.5 mg/ml in methanol. Linear calibration curves obtained for BPA ranged from 3.9-250 ng/ml, and the coefficients of the determinations (r^2^) were ≥ 0.995. The QA/QC materials were prepared from a plasma pool obtained from multiple anonymous pregnant women donors in analysis with standard, reagent blank, and unknown samples. We performed external calibration using the chromatographic responses of seven standard concentrations in their corresponding solvent. The recovery rates of blanks extended from 96-103%. The relative standard deviation (RSD) among triplicate analyses were 1.99-7.53%, and the recovery percentage was 96.1% with an RSD of 7.53%. Concentrations below the limit of detection (LOD) of 0.13 ng/ml [n = 25 (26%)] were given a value of LOD/√2 for statistical analyses [21].

### Enzyme-linked immunosorbent assay (ELISA)

The concentrations of plasma adiponectin and leptin protein were determined using Quantikine^® ^human immunoassay kits (R&D Systems Inc, Minneapolis, MN) according to the operation manual. A 96-well polystyrene microplate was coated with mouse monoclonal antibodies individually against adiponectin or leptin globular domain conjugated to horseradish peroxidase. After incubation, stabilized hydrogen peroxide and chromogen tetramethylbenzidine were added, and the optical density of each well was determined at 450 nm using a VERSAmax microplate reader (Molecular Devices, Sunnyvale, CA). The levels of adiponectin and leptin were deduced from the absorbance value by extrapolation from a standard curve generated in parallel. The minimum detectable dose of adiponectin and leptin is typically less than 0.246 ng/ml and 7.8 pg/ml.

### Birth outcome

The newborns were assessed as (a) low birth weight (LBW) defined as a newborn's birth weight less than the 10th percentile (< 2, 600 g; n = 20); (b) small for gestational age (SGA) defined as birth weight less than the 10th percentile, compared with the birth weight distribution in the same gestation week and gender according to the data of nationwide newborn's birth weight percentiles by gestational age in Taiwan [22]; (c) high leptin (HLP) defined as the level of leptin more than 90th percentile (> 9.56 ng/ml; n = 25) in cord blood samples; and (d) low adiponectin (LAD) defined as the level of adiponectin less than 10th percentile (< 10.32 μg/ml; n = 22).

### Statistical analysis

To consider potential confounding variables, a multivariable logistic regression was used to model the odds of adverse outcome variables (LBW, SGA, HLP and LAD). Previous studies have suggested that maternal metabolic parameters (HDL, TC, TRG, leptin and adiponectin) are important confounders relevant to birth weight and adipokine levels [23,24]. Thus, other than maternal age, BMI, serum BPA concentration, smoking and socioeconomic status, the maternal metabolic parameters, i.e., HDL, TC and TRG were selected as the independent variables for newborn LBW and SGA outcomes. Moreover, the maternal concentrations of leptin and adiponectin were included as potential confounders for cord blood leptin and adiponectin concentrations.

All statistical analyses were conducted using the SPSS statistical package (SPSS version 18.0, Chicago, IL). For continuous exposure variables, a categorical analysis was performed to compare the difference of study subjects based on BPA exposure tertile of pregnant women for testing dose response. We used Spearman correlation coefficients to assess the correlation of birth outcomes with categories of the BPA exposure level in pregnant women. Statistical differences were analyzed using the Mann-Whitney *U *test for nominal variables, and Student's *t-*test or one-way analysis of variance (ANOVA) for continuous variables. Multiple logistic regression was used to evaluate the odds ratio of potential effects on birth outcome attributed to maternal BPA concentration. All statistical significances were determined with a two-tailed *p *< 0.05.

## Results

**Figure 1:**
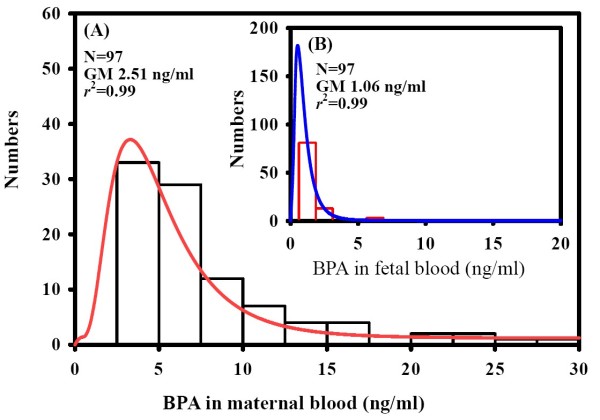
**Distribution of BPA concentrations in (A) maternal blood samples and (B) fetal umbilical cord blood**.

Figure 1A and Figure 1B showed the distributions of BPA levels respectively in the 97 maternal blood samples and the 97 fetal umbilical cord blood samples. A log normal distribution was used to fit both the maternal and fetal BPA concentrations, which display high fitness (*r*^2 ^= 0.99). Maternal BPA concentrations were highly variable (ranging from 0.3 to 29.4 ng/ml), whereas most fetal BPA levels were < 1 ng/ml (77% of the samples were 0.3 ng/ml). The geometric means (GM) of maternal and fetal BPA levels were 2.5 ng/ml and 0.5 ng/ml, respectively. The GM of maternal BPA levels was five-fold higher than the fetal umbilical cord blood.

Table 1 presented demographic characteristics, BPA levels, and lipid profiles of the 97 pregnant women and their newborns who participated in this study. The average (± S.D.) age, height, weight and BMI of pregnant women were 28.8 ± 3.7 years, 160.9 ± 5.2 cm, 65.3 ± 11.2 kg, and 25.2 ± 4.0 kg/m^2^. The average values for serum BPA, adiponectin and leptin were 5.4 ± 6.3 ng/ml, 6.6 ± 4.7 μg/ml, and 17.9 ± 13.9 ng/ml in pregnant women, and 1.1 ± 2.2 ng/ml, 21.3 ± 8.2 μg/ml, and 4.6 ± 3.5 ng/ml in neonates. The neonates average weight and gestational age were 3109.6 ± 319.0 g and 38.9 ± 1.1 weeks, respectively. Gender distribution among neonates was 57.7% male and 42.3% female.

**Table 1:** Baseline anthropometric, metabolic characteristics and levels of adipokines corresponding to BPA level in study subjects

		Group^a^		
		
Variables	All (n = 97)	High BPA (n = 62)	Low BPA (n = 35)	*p*^d^
*Anthropometric characteristics*				
*Pregnant women*				
Age (years)	28.8 ± 3.7	29.2 ± 2.9	29.1 ± 3.9	0.43
Height (cm)	160.9 ± 5.2	158.7 ± 4.7	161.9 ± 5.1	0.31
Weight (kg)	65.3 ± 11.2	64.2 ± 11.0	66.7 ± 11.5	0.35
BMI (kg/m^2^)	25.2 ± 4.0	25.4 ± 3.6	25.5 ± 4.3	0.85
Smoking				0.69
Yes	12 (13%)	8 (13%)	4 (11%)	
No	85 (87%)	54 (87%)	31(89%)	
Alcohol				0.78
Yes	7 (7%)	4 (6%)	1 (3%)	
No	90 (90%)	58 (94%)	34 (97%)	
Socioeconomic status				0.97
High	26 (27%)	16 (26%)	9 (26%)	
Medium	24 (25%)	10 (18%)	8 (23%)	
Low	47 (48%)	35 (56%)	18 (51%)	
TC (mg/dl)	218.9 ± 80.5	187.3 ± 101.2	232 ± 68.4	**< 0.05**
TRG (mg/dl)	144.4 ± 54.9	142.3 ± 62.4	145.2 ± 52.8	0.59
HDL (mg/dl)	42.9 ± 13.6	47.3 ± 15.0	41.3 ± 13.0	0.36
LDL (mg/dl)	148.5 ± 72.1	115.6 ± 93.7	161.4 ± 59.3	**< 0.05**
BPA level (ng/ml)^b^	5.4 ± 6.3	11.7 ± 6.4	2.1 ± 1.6	**< 0.01**
Adiponectin protein (μg/ml)	6. 6 ± 4.7	6.4 ± 3.9	6.8 ± 5.2	0.85
Leptin protein (ng/ml)	17.9 ± 13.9	17.6 ± 12.5	18.6 ± 14.9	0.81
*Neonates*				
Birth weight (g)	3109.6 ± 318.9	3067.9 ± 356.4	3212.9 ± 241.2	0.13
Gestation age (weeks)	38.9 ± 1.1	38.9 ± 1.1	38.6 ± 1.2	0.57
Infant sex				0.18
Male	56 (57.7%)	32 (51.6%)	24 (68.6%)	
Female	41 (42.37%)	30 (48.4%)	11 (31.4%)	
BPA level (ng/ml)^c^	1.1 ± 2.2	0.5 ± 0.6	1.4 ± 2.9	0.12
Adiponectin protein (μg/ml)	21.3 ± 8.2	22.1 ± 8.6	21.1 ± 8.2	0.53
Leptin protein (ng/ml)	4.6 ± 3.5	4.6 ± 4.1	4.7 ± 3.3	0.73

Values were presented as mean ± SD.

Abbreviation: TC (total cholesterol), TRG (triglyceride), HDL (high density lipoprotein) and LDL (low density lipoprotein), BMI (body mass index when delivery)

^a^Divided by geometric mean of maternal BPA level (2.51 ng/ml)

^b^BPA level was detected in the serum of pregnant women

^c^BPA level was detected in umbilical cord serum

^d^*p*-value from Mann-Whitney U test

Based on the GM of maternal BPA levels, study subjects were divided into high- and low-BPA groups. The levels of TC and LDL were significantly higher in the low-BPA group than in the high-BPA group. Additionally, no significant differences in TRG, HDL, adiponectin and leptin levels were observed between these two groups. Neonates in the high BPA group were found to have lower birth weights than those in the low BPA group (*p *= 0.13). Some relevant differences between these two BPA groups were related to the gender of the infants.

To examine whether BPA exposure in pregnant women affected fetal BPA exposure and adverse outcomes, we analyzed the correlation between maternal BPA level, lipid profile, adipokines, birth weight, and gestational age (Table 2). The maternal BPA levels showed a significantly inverse relationship to BMI (*r *= -0.48, at 3rd tertile BPA level), TC (*r *= -0.67, at 2nd tertile BPA level), and LDL (*r *= -0.61, at 2nd tertile BPA level), but were positively associated with TRG (*r *= 0.44, at 1st tertile BPA level). Moreover, the level of leptin displayed a significant correlation coefficient with increased BPA levels (*r *= 0.39 at 1st tertile, *r *= 0.50 at 2nd tertile, and *r *= 0.59 at 3rd tertile BPA level). Conversely, maternal BPA exposure had a significant negative association with neonatal BPA level (*r *= -0.53 at 3rd tertile BPA level) and birth weight (*r *= -0.24 at total sample and *r *= -0.39 at 3rd tertile BPA level).

**Table 2:** Correlation between serum BPA levels and characteristics of pregnant women and healthy neonates

	Spearman correlation coefficient			
	
Characteristics	BPA level (total sample)	BPA level (1st tertiles)	BPA level (2nd tertiles)	BPA level (3rd tertiles)
*Pregnant women*				
Age (years)	0.07	0.01	-0.03	0.12
BMI (kg/m^2^)	-0.08	-0.01	-0.21	-0.48*
TC (mg/dl)	-0.12	0.29	-0.67*	0.29
TRG (mg/dl)	0.20	0.44*	-0.52	0.45
HDL (mg/dl)	**0.33^#^**	0.33	-0.21	-0.19
LDL (mg/dl)	-0.24	0.19	**-0.61^#^**	0.29
Adiponectin (μg/ml)	-0.07	-0.19	-0.02	-0.09
Leptin (ng/ml)	0.11	0.39*	0.50*	0.59*
*Newborns*				
BPA level (ng/ml)	-0.05	-0.02	-0.32	-0.53*
Birth weight (g)	-0.24*	-0.04	-0.24	**-0.39^#^**
Gestation age (weeks)	0.05	0.19	0.09	-0.31
Adiponectin (μg/ml)	0.06	0.19	0.26	0.21
Leptin (ng/ml)	-0.08	0.09	0.05	0.04

BPA level divided into three quartiles: Q1 0.3-3.38 (ng/ml), Q2 3.38-7.04 (ng/ml), Q3 > 7.04 (ng/ml)

^#^*p-*value < 0.1, *p-value < 0.05

As shown in Table 3, the crude ORs showed that maternal BPA exposure was associated with LBW (OR 2.65, 95% CI 1.43-3.47) and SGA (OR 1.93, 95% CI 1.44-2.56) for male neonates but was not significantly associated with fetal adiponectin and leptin. Further adjustment for BMI, TC, TRG, HDL, LDL and adipokines attenuated this association, but maternal BPA exposure was still significantly related to an increased risk of fetal LBW (OR 2.12, 95% CI 1.05-2.38) and SGA (OR 1.34, 95% CI 1.13-2.83) in males. Additionally, the ORs of fetal adverse outcomes and the maternal BPA level were not observed in female neonates.

**Table 3:** Odds ratio of BPA level contributed to low birth weight, small for gestation age, high leptin level, and low adiponectin level in neonates

Conditionsa	Infant/Mother Pairs	No. of cases	Crude OR (95% CI)^a^	Adjusted OR (95% CI)^a^
LBW				
All	96	96	3.87 (1.03-4.51)	3.49 (1.07-4.36)^b^
Male	69	69	2.65 (1.43-3.47)	2.12 (1.05-2.38)^b^
Female	27	27	1.95 (0.51-3.44)	1.81 (0.42-2.09)^b^
SGA				
All	97	97	2.99 (1.18-5.59)	1.84 (1.55-2.24)^b^
Male	51	51	1.93 (1.44-2.56)	1.34 (1.13-2.83)^b^
Female	46	46	1.09 (0.31-3.85)	1.02 (0.57-2.09)^b^
LAD				
All	68	68	1.06 (0.28-2.97)	0.45 (0.07-1.06)^c^
Male	38	38	3.00 (0.50-4.95)	1.76 (0.99-3.13)^c^
Female	30	30	1.22 (0.02-2.22)	1.17 (0.01-2.13)^c^
HLP				
All	68	68	1.34 (0.47-3.83)	1.82 (0.35-2.54)^d^
Male	38	38	3.09 (0.56-5.17)	3.74 (0.27-6.74)^d^
Female	30	30	1.07 (0.23-2.01)	1.14 (0.49-2.29)^d^

Abbreviation: LBW (low birth weight), SGA (small for gestation age), LAD (low adiponectin level), HLP (high leptin level)

^a^Categorical analysis was performed to compare the odds ratio for every birth outcome based on geometric mean of maternal BPA exposure (2.51 ng/ml). The level of BPA less than LOD was recorded as 1/√2 LOD.

^b^Adjusted for maternal age, BMI, smoking and socioeconomic status, HDL, TC and TRG

^c^Adjusted for maternal age, BMI, smoking and socioeconomic status and adiponectin

^d^Adjusted for maternal age, BMI, smoking and socioeconomic status and leptin

**Figure 2:**
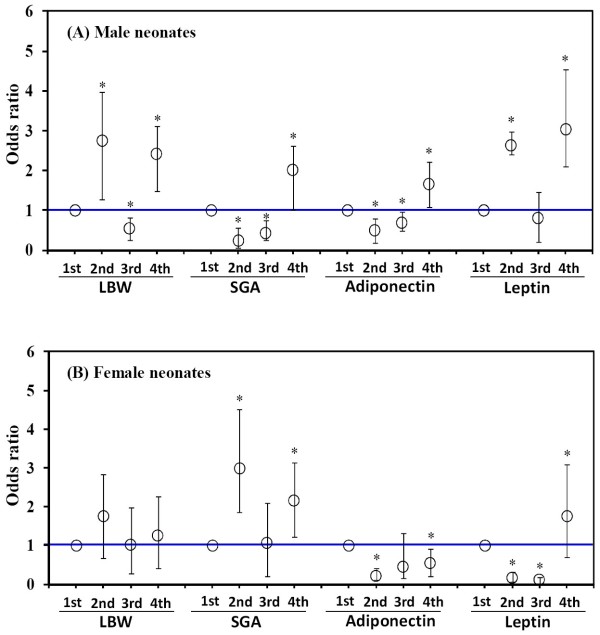
**Adjusted ORs (95% CIs) of LBW, SGA, HAD and LAP by maternal BPA exposure quartiles**. *Statistical significance at 5%.

The risks of fetal LBW, SGA, LAD, and HLP were plotted by quartiles based on maternal BPA exposure levels in Figure 2. Among male infants, the multivariable adjusted ORs of LBW of the second (OR 2.75, 95% CI 1.52-4.22) and fourth quartile (OR 2.42, 95% CI 1.72-3.36) of maternal BPA exposure, in contrast to the first quartile of BPA levels, showed that maternal BPA levels were significantly associated with an increased risk of LBW. The adjusted ORs of SGA of the second (OR 0.24, 95% CI 0.17-0.32) and the third quartile (OR 0.44, 95% CI 0.20-0.53) of maternal BPA level were significantly decreased, but increased in the fourth quartile (OR 2.01, 95% CI 0.99-0.62) (Figure 2A). The dose- response curve at SGA in male infants illustrated a U-shaped curve, but showed adverse effects at the highest BPA exposure (4th quartile). Additionally, significantly increased risks of LAD (OR 1.67, 95% CI 1.12-2.25) and HLP (OR 3.03, 95% CI 2.09-4.54) were observed for male neonates at the highest BPA exposure quartile (Figure 2A). In female neonates, BPA levels in the second (OR 2.99, 95% CI 1.47-4.11) and the fourth quartile (OR 2.17, 95% CI 1.19-3.12) were positively correlated with an increased risk of SGA presenting a Z-shaped curve and adverse effects (Figure 2B). Otherwise, the ORs of LAD were significantly decreased in the second (OR 0.21, 95% CI 0.10-0.21) and the fourth exposure quartiles (OR 0.55, 95% CI 0.34-0.56). The ORs of HLP at the second (OR 0.18, 95% CI 0.12-0.18) and third quartile (OR 0.11, 95% CI 0.06-0.11) of maternal BPA exposure were significantly decreased, but increased in the fourth quartile (OR 1.75, 95% CI 1.04-1.85) (Figure 2B). The dose-response curve of HLP in female infants showed a U-shape, but adverse effects were observed at the highest exposure quartile of BPA.

## Discussion

As BPA recently has been clinically shown in human and animals to cross the placental barrier, the potential effects of maternal BPA exposure on prenatal development has become a more focal area of research [10,25]. Previous studies have shown that prenatal BPA exposure in rodents and human is associated with fetal developmental and metabolic adverse effects, for example, increased birth weight [19,26], reduced gestational age [27], and disrupted thyroid function [28]. These findings raise concerns as to the developmental toxicity since the active BPA dose is considerably lower than the reported concentrations in the serum of pregnant women in the U.S. (5.9 ng/ml) and in South Korea (2.7 ng/ml) [29,30]. This study is the first to show the correlation between maternal BPA exposure and birth outcomes in Taiwan. We analyzed BPA levels in blood samples obtained from pregnant women and umbilical cords. The levels of BPA ranged from 0.3 to 29.4 ng/ml (GM 2.5 ng/ml) and 0.3 to 18.5 ng/ml (GM 1.1 ng/ml) in pregnancy and cord serum, respectively. A recent study indicated that approximately 27% of BPA can readily cross a placenta [8] and that maternal BPA exposure transferred to the fetus can cause developmental abnormalities and other adverse health effects in the offspring [10-13]. This study found a 13% transfer of BPA from maternal blood to fetal cord blood. The results indicated that fetuses can be exposed to BPA *in utero *despite differences in the placental transfer percentage of BPA in a previous study [10]. In male neonates, this study found a significantly negative correlation between fetal birth weight and maternal BPA level. Additionally, the risks of LBW and SGA and higher fetal leptin and adiponectin levels increased with maternal BPA levels in the highest tertile. That prenatal exposure to BPA seems to cause adverse effects on fetal growth and development is an important concern.

The present study revealed several important findings. First of all, we found a correlation between maternal BPA exposure and birth outcomes. Prenatal BPA exposure in the highest quartile (> 7.04 ng/ml) was inversely associated with birth weight, whereas it was not significantly correlated at the lowest and second quartiles of BPA concentrations. Some animal studies supported our finding that the reduction in weight of both male and female offspring is associated with prenatal or postweaning exposure to BPA [12,31]. Kim et al. [32] reported that administration of a high BPA level (300 mg/kg) during the entire gestational period in Sprague-Dawley rats reduced the weight of the fetuses. Maternal exposure in sheep at BPA levels of 30 to 50 ng/ml during days 30 to 90 of gestation resulted in low birth weight in offspring [33]. However, certain studies provided conflicting results, reporting an increased weight in offspring whose mothers were exposed to BPA during gestation [26,34,35]. Moreover, mice embryos cultured at the two-cell stage in 1 nM or 100 μM BPA showed no noted differences in pup weight at birth [36]. However, at the time of weaning on postnatal Day 21, offspring from embryos exposed to either dose of BPA were significantly heavier than the control offspring. Although this study showed that male fetal birth weight was negatively correlated with a high level of maternal BPA exposure, the BPA treatment level of animal models compared to human exposure should be considered. Lee et al. [30] also indicated that maternal BPA levels have more marked effect on male fetuses than female fetuses, but they found a significant positive correlation between maternal serum BPA levels and fetal birth weight.

Estrogen is known to stimulate cell proliferation for growth and development. Troisi et al. [37] indicated that the serum estrogen level of pregnant women is positively correlated with fetus weight, fetus length, and head circumference, but with no existing correlation between the estrogen level of the umbilical cord and fetus weight. Nagata et al. [38] also found a positive association between birth weight and maternal serum estradiol and estriol levels. Aromatase converts testosterone to estradiol [39]. Prenatal testosterone treatment leads to growth retardation and compromised estradiol-positive feedback, as illustrated by Manikkam et al. [40], leading to growth retardation and consequently resulting in low birth weight in sheep. Savabieasfahani et al. [33] reported low birth weight in offspring because of prenatal exposure to BPA and suggested that the effect may be facilitated through a conversion of testosterone to estradiol. In the present study, the prenatal BPA exposure in male fetuses with low birth weight suggested a response to testosterone feedback. Thus, the magnitude of BPA effect on weight may be influenced by subtle changes of hormones *in utero*.

Second, our data demonstrated that pregnant women exposed to BPA levels greater than 2.51 ng/ml had a higher risk of giving birth to males with LBW (OR 2.12, 95% CI 1.05-2.38) (Table 3). Moreover, in the male offspring, the lower and highest quartiles of BPA levels conferred a greater risk of LBW than the lowest quartile of BPA level (Figure 2A). This finding produced a non-monotonic or a U-shaped dose-response curve consistent with the previous report [41]. Additionally, male neonates suffered an approximately 34% higher risk of SGA from a maternal BPA level higher than 2.5 ng/ml (Table 3). A linear dose-dependent response was noted at increased quartiles of BPA levels (Figure 2). Conversely, the secretion of leptin and adiponectin was used as a predictor to assess the potential risk of developing metabolic syndrome in newborns. Additionally, a higher relative risk of increased leptin secretion in both male and female infants corresponding to pregnant women exposed to the highest quartile BPA level (Figure 2). As mentioned earlier, our results implied that the effects of maternal BPA exposure produced a U-shaped dose and gender dependence on fetal birth outcomes. These results were consistent with several published studies. Differential processing of high dose BPA relative to low dose contributes to the U-shaped, non-monotonic dose-response curve and gender-difference effects [42,43]. Sex- and dose-dependent differences in weight in response to early postnatal exposure to diethylstilbestrol, a synthetic estrogen similar to the BPA structure, have been reported [35]. Prenatal or neonatal exposure to BPA was correlated with adverse effects on fetal growth parameters such as LBW and SGA. Manikkam et al. [40] reported that exposure to endocrine-disrupting compounds *in utero *caused fetal growth retardation in sheep. Our data suggests that BPA mimics estrogen in its action, and continued exposure to BPA during gestation is likely to have an impact on the fetus' developmental trajectory. In fact, a growing body of evidence, in addition to our data, had considered LBW and SGA to be a potential outcome of fetal exposure to BPA or endocrine-disrupting compounds [33,35].

Thirdly, our data showed that prenatal exposure to BPA may lead to a potential risk of altering metabolic features in the fetus such as increasing adverse secretion of letpin and adiponectin. Phrakonkham et al. [44] suggested that BPA increases gene expression of adipogenic transcription factors in 3T3-L1 preadipocytes. Perinatal BPA exposure is associated with the over-expression of adipocyte hypertrophy and of lipogenic genes in rats [12]. Moreover, BPA was reported to inhibit adiponectin secretion from human adipocyte explants in a dose-dependent manner [45]. An *in vivo *study suggested that neonatal estrogenization can influence fetal development including fetus weight [28]. These studies provided indirect evidence supporting our observation of BPA altering the secretion of fetal adipokines. However, more information about the adipogenic effect of BPA in fetuses is required for confirmation.

This study has several limitations. First, the level of plasma BPA was only detected at a single time in connection with delivery. Some imprecision exists because BPA exposure is variable over time [46]. Thus, this study assumed that the BPA exposure sources, for example, living environment, consumption habits, and exposure routes, were sustained, and that the exposure level of BPA influencing fetal development and health would be persistent in maternal plasma, though the half-life of BPA is relatively short. Second, the statistical modeling included only maternal age, BMI, and metabolic parameters as control variables to adjust for birth outcomes (LBW and SGA), but parity, a potential confounding factor for birth weight, was not considered. Additionally, it would be reasonable to concede from the results of this study that maternal BPA levels were not significantly correlated with fetal BPA levels. Nishikawa et al. [47] reported that maternal BPA-glucuronide (BPA-GA) may cross through the placenta and deconjugate to BPA in the fetus. Our results suggest that prenatal exposure to BPA might influence fetal development even though we found no significant correlation was present between maternal and fetal BPA levels due to the differences in the drug-metabolizing system of mother and fetus. However, these inferences still require further confirmation.

## Conclusions

The BPA levels detectable in cord blood indicated that BPA can be transported to the fetus across the placental barrier. While earlier animal studies have shown a linkage between BPA exposure and adverse fetal effects, this study quantified and illustrated a potential risk in human neonates. However, obtaining more samples from pregnant women and fetuses under varying exposure levels is necessary to reliably assess the risk posed by BPA to humans.

## List of Abbreviations

BPA: Bisphenol A; HLP: secretion of high leptin; HPLC: high performance liquid chromatography; LBW: lower birth weight; LAD: secretion of low adiponectin; SGA: small for gestation age; LOD: limit of detection; TC: total cholesterol; TRG: triglyceride; HDL: high density lipoprotein; LDL: low density lipoprotein; BMI: body mass index.

## Competing interests

The authors declare that they have no competing interests.

## Authors' contributions

WCC performed the statistical analysis and wrote the manuscript. JLC was involved in processing the raw data and blood sample collection. CFL conducted the determination of blood BPA and adipokines. YCC designed and tested the power and validity of questionnaires, and trained the interviewers. FCS was responsible to contact the study subjects. CYC directed the studies and revised the manuscript. All the authors contributed to the discussion of data and approved the final manuscript.
